# Assessment of toxic elements in sediments of Linggi River using NAA and ICP-MS techniques

**DOI:** 10.1016/j.mex.2018.05.001

**Published:** 2018-05-17

**Authors:** Md Suhaimi Elias, Shariff Ibrahim, Kamarudin Samuding, Shamsiah Ab Rahman, Yii Mei Wo

**Affiliations:** a School of Chemistry and Environment, Faculty of Applied Sciences, Universiti Teknologi MARA (UiTM), 40450, Shah Alam, Selangor, Malaysia; b Analytical Chemistry Application Group (ACA), Waste and Environmental Technology Division, Malaysian Nuclear Agency, Bangi, 43000, Kajang, Selangor, Malaysia; c Environmental Tracer Application Group (E-TAG), Waste and Environmental Technology Division, Malaysian Nuclear Agency, Bangi, 43000, Kajang, Selangor, Malaysia; d Radiochemistry and Environmental Group (RAS), Waste and Environmental Technology Division, Malaysian Nuclear Agency, Bangi, 43000, Kajang, Selangor, Malaysia

**Keywords:** Linggi River, Enrichment factor, Degree of contamination, Freshwater sediment quality guidelines, NAA, ICP-MS

## Abstract

Fourteen sediment samples were collected along Linggi River, Malaysia. Neutron activation analysis (NAA) and inductively coupled plasma-mass spectrometry (ICP-MS) techniques were used in the determination of toxic element contents. The results showed that As, Cd and Sb concentrations were higher at all sampling stations, with enrichment factor values ranging from 17.7 to 75.0, 2.1 to 19.5 and 6.6 to 28.4, respectively. Elements of Pb and Zn) were also enriched at most of the sampling stations whilst Cu, Cr and Ni were shown as background levels. The sediment of Linggi River can be categorised as low (<8.0) to very high degree of contamination (>32.0). The mean concentrations of elements viz. Cd, Cr, Ni, Pb, Sb and Zn were lower than the threshold effect level (TEL) of FSQGs values except for As. The concentration of As (arsenic) was higher than PEL and PEC of FSQGs values.

## Graphical abstract

**Figure:**
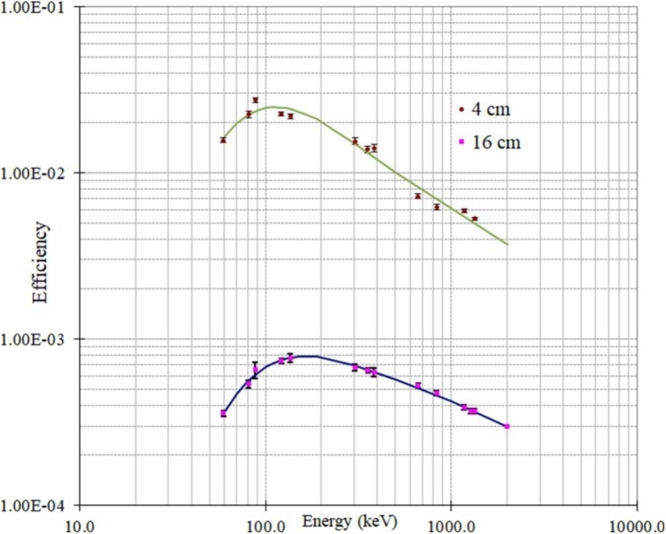

## Method details

### Background

Heavy metals are considered as serious inorganic pollutants, that can be accumulated in sediments and aquatic food chain [1,2]. These can give adverse effects to the aquatic life [3,4]. Water, sediment and biota significantly play an important role in the assessment of the level of pollution, degree of contamination and toxicology effects [[5], [6], [7]].The major sources of inorganic pollution originated from anthropogenic sources are industrial, domestic, animal waste, mining, petroleum activities and agriculture activities, as well as industrial emissions [8,9].

Sediment Quality Guidelines (SQGs) were developed to assist regulator and enforcement to mitigate and dealing with the contaminated sediment [10]. Typically, the total concentrations of contaminants in the whole sediments are compared to the guideline values to determine whether there is a potential for benthic invertebrate community impairment [11]. SQGs can be used in environmental assessments in combination with other measures such as the water quality, the concentration level and the degree of contamination to evaluate the risk to aquatic ecosystems from the anthropogenic activities. In this study, consensus-FSQGs and Canadian-FSQGS concentration values were used as a reference to evaluate the risk of sediment concentration to the benthic and sediment-dwelling organisms.

Assessment of pollution level in water and sediment of the Linggi River is important since Linggi River supplies water to Seremban and Port Dickson area [12]. The Linggi River, was classified as class III by Department of Environment, Malaysia which has required extensive treatment for water resources [13]. The Linggi River pollution was reported on the elemental pollution in water and suspended sediment by Khan (1990). However, since then, there has been no recent work reported regarding heavy metal pollution in sediments collected from Linggi River. Study of heavy metal contents, degree of contamination and also compared with freshwater sediment quality guidelines (FSQGs) of the Linggi River are still limited. In the present study, toxic elements As, Cd, Cr, Cu, Ni, Pb, Sb and Zn in sediments were selected due to their importance with respect to public health concern and impact to the river ecosystem.

## Materials and methods

### Sampling locations

**Fig. 1:**
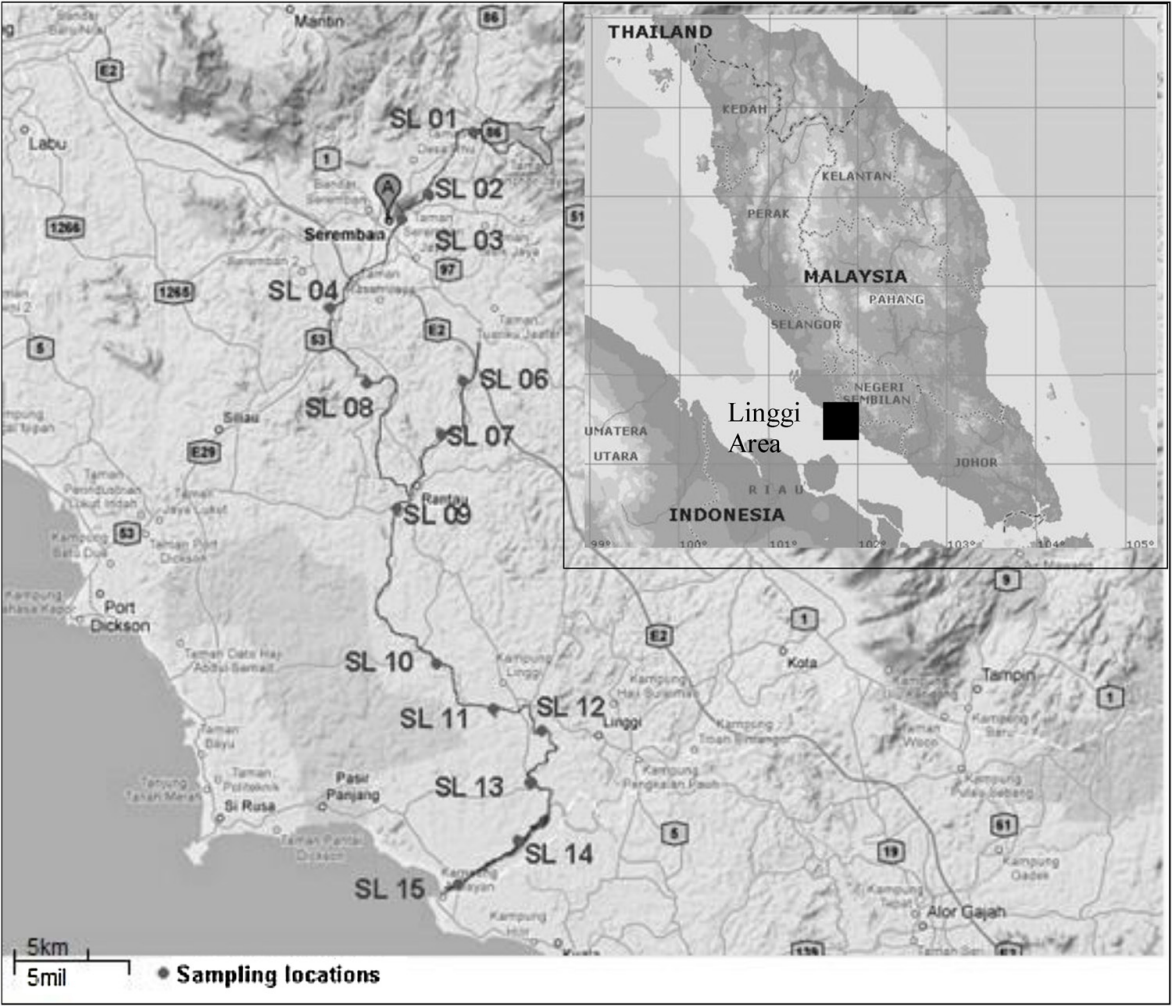
Map showing sampling locations.

Fourteen sampling locations were selected along the Linggi River as shown in Fig. 1. The surface sediment samples were collected by using a Ponar grab sampler. Sediment samples were kept in polyethylene bottle and transported to the laboratory. Sediments were dried in an oven at 60 °C until constant weight, ground to a powder form with an agate mortar and then sieved through 63 μm mesh sieve and kept in polyethylene containers.

## Preparation of mix standard solution, samples and standard reference material (SRM) prior irradiation

Single standard solutions of As, Sb, Cr, Zn and Fe were purchased from Merck brand. The mix standard solution was prepared in volume metric flask of 100 ml. The concentration of mix standard solution of As, Sb, Cr, Zn and Fe are 10, 10, 80, 100, and 1000 mg/L, respectively. Filter papers (Ø = diameter, 1 cm, Whatman brand) were inserted into small polyethylene vial (Ø 1 cm × 3 cm H). An aliquot of 0.2 mL (∼0.2 g) of mix standard solution was dropped onto filter papers and it was dried at 50°C in an oven for 24 h. Polyethylene vial containing of mix standard solution was sealed using heating solder. Wet sediment samples from Linggi River were dried in an oven at 60 °C until constant weight, ground to a powder form with an agate mortar and then sieved through 63 μm mesh sieve and kept in polyethylene containers. The standard reference material (SRM) (IAEA-soil-7) were purchased from IAEA. Approximately 0.15 to 0.20 g homogenised sediment samples and SRM-IAEA-Soil-7 were weighed using the analytical balance into polyethylene vials and sealed with heating solder prior to the irradiation process [14].

## Irradiation process

The duplicate samples, mix standard solution and SRM were irradiated together in the 750 kW PUSPATI TRIGA Mark II reactor at Malaysian Nuclear Agency, with a thermal flux of 4.0 × 10^12^ n cm^–2^ s^–1^ [15]. The isotope of radionuclied and other information were shown in Table 1. The irradiation process of up to 6 h at the rotating rack for the long-life radionuclides (As, Sb, Cr, Zn and Fe) were performed. Cooling time for a decay process were ranged from 2 to 4 days before performing the first counting, and 3 to 4 weeks for the second counting using gamma spectrometry.

**Table 1:** The elements and radionuclied measured using Neutron Activation Analysis (NAA) technique.

Procedure	Elements	Radionuclied	Half-time	γ-ray Energy (keV)
Irradiation: 6 h. Counting time: 1 h Cooling time: 2 – 4 days	As	^76^As	26.4 hours	559
	Sb	^122^Sb	2.70 days	564
	Sb	^124^Sb	60.9 days	603
	Cr	^51^Cr	27.8 days	320
	Zn	^65^Zn	244 days	1115
	Fe	^59^Fe	45.1 days	1099, 1292

## Counting process and concentration measurement by using NAA technique

**Fig. 2:**
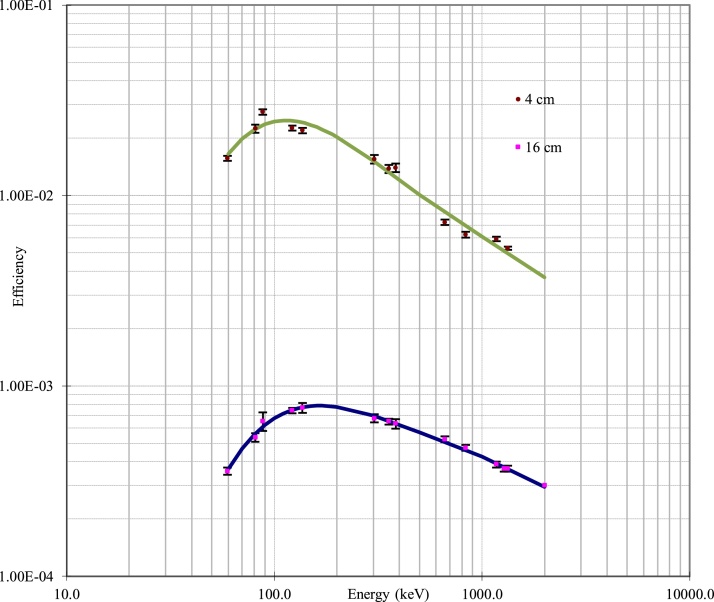
The efficiency curve of gamma ray calibration of gamma spectrometer at different geometry.

The counting process of the irradiated samples, mix standard solution and SRM were performed for one hour each, by using gamma spectrometry. The detector was calibrated from low to high energy of gamma ray by using ^241^Am (59.5 keV), ^109^Cd (88.1 keV), ^57^Co (122.1, 136.5 keV), ^133^Ba (81.0, 303.0, 356.0, 384.0 keV), ^137^Cs (661.7 keV), ^60^Co (1173.2, 1332.5 keV) and ^88^Y (898.0, 1836.1 keV)[16,17]. The efficiency curve of gamma ray calibration of gamma spectrometer was shown in Fig. 1. The counting geometry of samples, mix standard solution and SRM were performed at 16 cm and 4 cm for the fist and second counting, respectively. The signal of γ-ray from the respective energy of elements were detected by a coaxial hyperpure germanium detector supply by EG&G ORTEC. The signals were then amplified and connected to multichannel analyser (MCA) and then the signal was converted to the photopeak (as net count area) by Gamma Vision software. Computations of elemental concentrations were based on comparative method and data were reported in dry weight (d.w.). The concentration of the sample and SRM was measured by using Eq. (1) [15,18] (Fig. 2).

(1) $$C_{El}=\frac{A_{smp}}{A_{std}}*\frac{W_{std}}{2_{smp}}*C_{std}$$

Where: A_smp_= net count of the selected peak area of an interested element in a sample

A_std_= net count of the selected peak area of an interested element in a standard

W_smp_= Weight of sample used

W_std_= Weight of standard used

C_std_= Concentration of interested element in standard (e.g.: μg/g, mg/kg)

C_El_= Concentration of interested element in sample (e.g.: μg/g, mg/kg)

## Digestion of sediment sample

Approximately 0.1–0.2 g of homogenised sediment samples of Linggi River were digested in containing of 5 mL nitric acid (67% HNO_3_ – TraceMetal Fisher brand) and 2 mL concentrated hydrofluoric acid (49% HF – analytical grade, QRëC^®^ brand) by microwave oven (Mars 5 brand). Each digestion batch was included at least two reagents blank acid, SRM (e.g., IAEA Soil-7) and duplicate samples. The microwave oven was programmed as follows: The power of the microwave setup at 1200 W, temperature setup for 200 °C, ramped for 20 min, held for 15 min, pressure setup at 0.6 MPa [7]. Sediment samples were digested for 20 min. After cooling for at least 30 min and microwave temperature below 50 °C, samples were removed from the microwave oven. If the solutions contained some residue, 1 mL HNO_3_ was added and the digestion process was repeated until clear solutions were obtained. After that, the solution was transferred into Teflon beaker and rinsed with 3 mL Milli-Q water. The Teflon beaker containing the digested sample was heated at 60 °C to 70 °C on a hot plate until dry and the Teflon beaker was then rinsed with 20 mL Milli-Q water. The solution was then filtered with filter paper (Ø=diameter 125 mm, whatmann brand) into a polyethylene bottle and lastly brought up to a volume of 50 ml with Milli-Q water for ICP-MS analysis.

## Analysis of samples using inductively coupled plasma – mass spectrometry (ICP-MS)

The commercial mix standard solution (Standard^®^ X) was purchased from Perkin Elmer. Instrumental operating conditions and data acquisition settings of ICP-MS was shown in Table 2. The mix standard solution of 10, 50, 100 and 150 μg/L were prepared for standard calibration curve. The calibrations of Pb, Cd, Cu and Ni showed good linearity with a correlation coefficient (R^2^) >0.9998 (Table 2). The concentrations of Pb, Cd, Cu and Ni in the samples were analysed by ICP-MS (Perkin Elmer-Elan 6000). The reagent blank acid used in digestion process was monitored throughout the analysis and used to correct the analytical results.

**Table 2:** Instrumental operating conditions and data acquisition settings of ICP-MS.

Mode of operation	Operation condition
Vacuum pressure (standby)	2.8 × 10^−6^ Torr
Vacuum pressure plasma	2.5 × 10^−5^ Torr
Nebulizer Gas Flow	0.98 L/min
Lens Voltage	6.3 Volts
ICP RF Power	1075.0 Watts
Analog Stage Voltage	−2150.0 Volts
Pulse Stage Voltage	1100.0 Volts
Mass detection Correlation coefficient (R^2^) value	^111^Cd, ^63^Cu, ^60^Ni and ^208^Pb 0.999929 (^111^Cd), 0.999875 (^63^Cu), 999,920 (^60^Ni) and 0.999827 (^208^Pb).

## Quality control and quality assurance of analytical method

The SRM (IAEA Soil-7) was used as quality control and quality assurance in the analytical method analysis. The SRM measurement followed the same procedure as a sample analysis. The certified and measured value, percentage of recovery, coefficient of variation (CV) and other information are tabulated in Table 2. The recovery and coefficient of variation percentage of the analysed SRM ranged from 81.5 to 113.4% and 4.0 to 22.1, respectively (Table 3). The calculation of relative bias (%) and *U* test score are described in Eqs. (2) and (3), respectively. The calculated *U* test value is compared with critical value listed in the t-statistic table to determine if the analysed result differs significantly from the certified value at a given level of probability (Table 4). The *U* test score are acceptable with *U* test value is ≤1.95 [19].

(2) Relative−bias=Canalysed−CcertifiedCcertifiedX100%

(3) $$U-test_{}=\frac{\left|C_{analysed}-C_{certified}\right|}{\sqrt{(\sigma_{analysed}^{2}+\sigma_{certified}^{2}}}$$

Where:C_analysed_ = concentration of analysed value

**Table 3:** Certified and analysed values of standard reference material (SRM) IAEA-Soil-7 and other information.

Element	SRM (IAEA-Soil-7) Certified value (mg. kg^−1)^	SRM (IAEA-Soil-7) Analysed value (mg.kg^−1^)	Recovery (%)	Relative-bias (%)	U-test Score	Coefficient of Variation (%)	Limit of detection
Analysed by NAA							
As	13.4 ± 0.8	14.5 ± 1.0	108.2	8.2	0.86	6.8	0.05 μg/g
Cr	60 ± 14	57.9 ± 5.0	96.5	−3.5	0.14	8.6	1.0 μg/g
Fe	25700 ± 600	25950 ± 1100	101.0	1.0	0.20	4.2	0.01 %
Sb	1.7 ± 0.3	1.81 ± 0.40	106.7	6.7	0.22	22.1	0.05 μg/g
Zn	104 ± 9	118 ± 2	113.4	13.4	1.52	1.7	10 μg/g
Analysed by ICP-MS							
Cd	1.3 ± 0.2	1.40 ± 0.1	107.7	7.7	0.45	7.1	5.0 ng/L
Cu	11.0 ± 2.0	12.4 ± 0.5	112.7	12.7	0.68	4.0	50.0 ng/L
Pb	60.0 ± 11.0	50.0 ± 5.0	83.3	−16.7	0.83	10.0	20.0 ng/L
Ni	26.0 ± 5.0	21.2 ± 1.0	81.5	−18.5	0.94	4.7	20.0 ng/L

**Table 4:** The condition *U* test score and status of analysed result differs significantly from the certified value.

Condition (score)	Status
U-test < 1.64	The analysed result does not differ significantly from the certified value.
1.95 > U-test > 1.64	The analysed result probably does not differ significantly from the certified value.
2.58 > U-test > 1.95	It is not clear whether the analysed result differs significantly from the certified value.
3.29 > U-test > 2.58	The analysed result is probably significantly different from the certified value.
U-test > 3.29	The analysed result is significantly different from the certified value.

C_certified_ = concentration of certified value

σ_analysed_= standard deviation of analysed value

σ_certified_= standard deviation of certified value

## Distributions and concentrations of samples

The concentrations of toxic elements in the surface sediments from Linggi River are listed in Table 5. The mean concentrations of As, Cd, Sb, Pb and Zn were found to be 21.2, 2.9, 6.1, 2.1 and 1.6 times greater, respectively, than the continental crust (CC) values, whilst other elements such as Cu, Cr, Fe, and Ni showed lower concentrations as compared to CC values. All sampling locations of the study area showed higher concentration values of As compared to the CC value. The contamination of As, Cd, Sb, and Zn, were suspected originate from industrial activities. The concentrations of Cu, Pb and Zn concentration values were recorded higher at the downstream area of the Linggi River and were originated from the industrial effluents [12]. Most of the high concentrations of As, Cd, Sb, and Zn were observed at sampling locations of SL 09 – SL15, which are significantly derived from elementals pollution from Linggi River (main river) and its tributary (Simin river) (Fig. 1). High concentrations of Pb were observed at SL02 and SL03 (Seremban area), SL07 and SL09 (Rantau area), where most of the industrial activities are located. The contamination of Pb was suspected from industrial activities with effluents from metal smelting, electroplating and factories. However, the possible sources of As and Pb elements are originated from phosphate fertilizer, lead-arsenate insecticides, and pesticides used in the agriculture activities [20].

**Table 5:** The concentrations of toxic elements (mg/kg) in Linggi River sediments.

Location	As^*^	Cd^#^	Cr^*^	Cu^#^	Fe^*^	Ni^#^	Pb^#^	Sb^*^	Zn^*^
SL01	3.6	0.09	3.8	1.5	5160	3.4	15.8	0.24	20.1
SL02	15.4	0.16	8.2	3.4	13930	6.9	37.4	0.80	58.8
SL03	12.8	0.19	7.2	2.9	13370	4.2	39.0	0.61	54.6
SL04	3.80	0.09	1.7	2.3	1990	1.8	8.2	0.26	12.4
SL06	29.9	0.15	26.0	14.5	17060	10.2	13.4	1.53	36.8
SL07	29.9	0.23	40.0	21.6	15940	14.0	52.3	3.14	171
SL08	36.5	0.42	21.5	11.0	12370	7.1	25.5	1.09	53.3
SL09	57.9	1.10	105	66.2	32040	29.7	46.0	4.44	430
SL10	57.1	0.45	47.3	21.3	22440	13.7	41.7	2.29	113
SL11	27.1	0.27	23.0	10.6	11670	9.1	27.9	1.45	67.0
SL12	45.2	0.34	37.4	14.7	18930	12.3	38.7	2.27	90.6
SL13	54.0	0.11	39.6	7.3	22610	6.3	13.6	2.25	90.1
SL14	65.6	0.21	49.5	13.7	28020	13.4	36.8	2.53	113
SL15	65.9	0.26	55.6	13.8	32410	12.7	30.2	2.65	130
Average	36.1	0.29	33.3	14.6	17710	10.3	30.5	1.83	103
CC	1.70	0.10	126.0	25.0	43200	56.0	14.8	0.30	65.0

CC = Continental crust values published by Wedephol (1995).

Sediment samples were analysed by ICP-MS (^#^) and NAA technique (*).

## Enrichment factor (EF)

In order to evaluate possible anthropogenic sources of toxic elements, the enrichment factor (EF) was calculated based on the Eq. (4) below:

(4) $$EF=\frac{{\left(M/R\right)}_{measure}}{{\left(M/R\right)}_{CC}}$$

Where M is the element of interest, R is the reference element, (M/R)_measure_ is the elemental ratio found in the sample, and (M/R)_CC_ is the elemental ratio in the continental crust. Iron (Fe) was used for normalisation purpose to determine the metal and heavy metal pollution of Linggi River. The selection of Fe as a normalisation element and used to be in the EF calculation was due to Fe distribution being not related to other heavy metals [21]. Fe usually has a relatively high natural concentration [22], and therefore not expected to be substantially enriched from anthropogenic source in estuarine sediment [23]. Most of the researchers suggested that EF values as the following: EF < 2 indicates no enrichment, EF = 2 to 3 is minor enrichment, EF = 3 to 5 is moderate enrichment, EF = 5 to 10 is moderately severe enrichment, EF = 10 to 25 is severe enrichment, EF = 25 to 50 is very severe enrichment and EF > 50 is extremely severe enrichment [24]. EF values of less than 2.0 indicate that the element in the sediment originated predominantly from lithogenous materials, whereas EFs are much greater than 2.0 indicating that the element is of anthropogenic origin[25].

The calculated EF values for selected elements of Linggi River are shown in Table 6. The EFs of As show enrichment in all sampling locations (EF values 17.7–75.0). Arsenic pollution can be categorised as severe to extreme enrichment. Cd, Pb, Sb and Zn can be categorised as minor to severe enrichment at most of the other sampling locations. Other elements such as Cr, Cu and Ni showed no enrichment at most of the sampling locations. EF of metals and heavy metals can be valuable and have been used as an indirect indicator for evaluation of sediment contamination or toxicity. However, it is not sufficient to use enrichment factor only for the evaluation of sediment toxicity at a particular site. Consideration for the degree of contamination in sediment and comparison with sediment guidelines are useful to evaluate the toxicity of the sediment for the particular site.

**Table 6:** Enrichment factor (EF) values of toxic elements in the sediments from Linggi River.

Location	As	Cd	Cr	Cu	Ni	Pb	Sb	Zn
SL01	**17.7**	**7.3**	0.3	0.5	0.5	**8.9**	**6.8**	**2.6**
SL02	**28.0**	**5.0**	0.2	0.4	0.4	**7.8**	**8.3**	**2.8**
SL03	**24.4**	**6.0**	0.2	0.4	0.2	**8.5**	**6.6**	**2.7**
SL04	**49.0**	**19.5**	0.3	2.0	0.7	**12.1**	**18.9**	**4.1**
SL06	**44.6**	**3.8**	0.5	1.5	0.5	**2.3**	**12.9**	1.4
SL07	**47.6**	**6.2**	0.9	**2.3**	0.7	**9.6**	**28.4**	**7.1**
SL08	**75.0**	**14.8**	0.6	1.5	0.4	**6.0**	**12.7**	**2.9**
SL09	**45.9**	**14.8**	1.1	**3.6**	0.7	**4.2**	**19.9**	**8.9**
SL10	**64.6**	**8.6**	0.7	1.6	0.5	**5.4**	**14.7**	**3.4**
SL11	**59.0**	**9.9**	0.7	1.6	0.6	**7.0**	**17.9**	**3.8**
SL12	**60.7**	**7.7**	0.7	1.3	0.5	**6.0**	**17.3**	**3.2**
SL13	**60.7**	**2.1**	0.6	0.6	0.2	1.8	**14.4**	**2.6**
SL14	**59.5**	**3.3**	0.6	0.8	0.4	**3.8**	**13.0**	**2.7**
SL15	**51.7**	**3.5**	0.6	0.7	0.3	**2.7**	**11.8**	**2.7**

Notes: Bold type indicates the enrichment of elemental pollution in sediment (EF value > 2.0).

## Degree of contamination (C_d_)

To describe the contamination of toxic elements in Linggi River, the following Eqs. 5 and 6 below are used to define as a contamination factor (C_f_) and degree of contamination (C_d_), respectively;

(5) $$C_{f}=\frac{C_{n}}{C_{0}}$$

(6) $$C_{d}=\sum_{i=1}^{n}C_{f}$$

where C_d_ is the degree of contamination, C_f_ is a contamination factor, *C_n_* is the metal content in the sediments and C_0_ is a background value (reference value of metals). The following terminology was used to describe the contamination factor: C_f_ < 1 low contamination factor (indicating low sediment contamination); 1 ≤ C_f_ < 3 moderate contamination factor; 3 ≤ C_f_ < 6 considerable contamination factor; C_f_ ≥ 6 very high contamination factor [26]. The contamination factor (C_f_) values were shown in Table 7. At all sampling stations As showed very high contamination factor, except at stations SL01 and SL04. Contamination factors of Cr, Cu and Ni can be categorised as low contamination. The elements of Cd, Pb, Sb and Zn can be categorised as low to very high contamination factor. The results indicated that contamination of sediments of Linggi River were mainly contributed by As, Cd, Pb, Sb and Zn.

**Table 7:** Contamination factor (C_f_) and degrees of contamination (C_d_) values of toxic elements from Linggi River.

Location	As	Cd	Cr	Cu	Ni	Pb	Sb	Zn	C_d_
	C_f_								
SL01	2.1	0.9	0.03	0.06	0.06	1.1	0.8	0.3	5.3
SL02	9.0	1.6	0.06	0.13	0.12	2.5	2.7	0.9	17.1
SL03	7.5	1.9	0.06	0.12	0.08	2.6	2.0	0.8	15.2
SL04	2.3	0.9	0.01	0.09	0.03	0.6	0.9	0.2	4.9
SL06	17.6	1.5	0.21	0.58	0.18	0.9	5.1	0.6	26.6
SL07	17.6	2.3	0.32	0.87	0.25	3.5	10.5	2.6	37.9
SL08	21.5	4.2	0.17	0.44	0.13	1.7	3.6	0.8	32.6
SL09	34.0	11.0	0.84	2.65	0.53	3.1	14.8	6.6	73.6
SL10	33.6	4.5	0.38	0.85	0.24	2.8	7.6	1.7	51.7
SL11	15.9	2.7	0.18	0.42	0.16	1.9	4.8	1.0	27.1
SL12	26.6	3.4	0.30	0.59	0.22	2.6	7.6	1.4	42.6
SL13	31.8	1.1	0.31	0.29	0.11	0.9	7.5	1.4	43.4
SL14	38.6	2.1	0.39	0.55	0.24	2.5	8.4	1.7	54.6
SL15	38.8	2.6	0.44	0.55	0.23	2.0	8.8	2.0	55.5

The degrees of contamination (C_d_) values of Linggi River are shown in Table 7. The degree of contamination (C_d_) is defined as the sum of all contamination factors (C_f_) of As, Cd, Cr, Cu, Ni, Pb, Sb, and Zn. Degree of contamination can be categorised into four categories according to the Hakanson (1980) classification. For the description of degree of contamination values, the following terminologies have been used: C_d_ < 8 low degree of contamination; 8 ≤ C_d_ < 16 moderate degree of contamination; 16 ≤ C_d_ < 32 considerable degree of contamination; C_d_ ≥ 32 very high degree of contamination. SL01 and SL04 can be categorised as low degree of contamination with C_d_ values 5.3 and 4.9, respectively. Sampling location of SL03 can be categorised as having a moderate degree of contamination. SL02, SL06 and SL11 stations can be categorised into considerable degree of contamination. Most of the sampling stations (eight stations) showed very high degrees of contamination with C_d_ values ranging from 32.6 to 73.6. This indicated very high loading of anthropogenic pollution at these eight sampling locations (SL07 to SL10 and SL12 to SL15).

## Comparison of toxic elements with FSQGs

In this paper, we adopted the Canadian Freshwater Sediment Quality Guidelines (Canadian-FSQGs) and the Consensus Freshwater Sediment Quality Guidelines (Consensus-FSQGs) published by MacDonald (2000) for the purpose of comparison between Malaysian rivers sediment and FSQGs. If a trace element concentration in sediment was less than the TEC or TEL values, the sediments were considered to be clean to marginally pollute. No effects on the majority of sediment-dwelling organisms were expected below the TEC or TEL concentration values. If the concentration of toxic element in sediment was greater than the PEC or PEL values, the sediments were to be considered heavily polluted. Adverse effects on the majority of sediment-dwelling organisms were expected when the concentrations exceeded PEC or PEL of FSQGs values.

Comparisons of toxic elements in sediments of the Linggi River and Malaysian rivers to Canadian FSQGs and Consensus FSQGs are shown in Table 8. Mean As concentrations of Linggi River in this study were higher than those of the Canadian-FSQGs – PEL value and Consensus-FSQGs – PEC value. These indicated that the Linggi River sediments were polluted with As and this may cause adverse effects to the majority of sediment-dwelling organisms. However, mean concentrations of Cd, Cr, Cu, Ni, Pb and Zn are less than the Canadian-FSQGs – TEL value and Consensus-FSQGs – TEC value. The concentration of As in sediments of Pelepah Kanan River, Kota Tinggi showed 4.5 times higher than the Consensus-FSQGs – PEC value and mean concentration of Cd in sediments of Langat River (12.1 mg/kg) showed higher concentration as compared to the Consensus-FSQGs – PEC value (4.98 mg/kg), as shown in Table 8.

**Table 8:** Comparison of toxic element concentrations in sediments of Malaysian rivers with freshwater sediment quality guidelines (FSQGs) (mg/kg d.w.).

Location	As	Cd	Cr	Cu	Ni	Pb	Sb	Zn
Linggi River, - present study (n = 14)	3.6 – 65.9 (36.0)	0.09 – 1.10 (0.29)	1.8 – 105 (33.2)	1.5 - 66.2 (14.6)	1.8 – 29.7 (10.3)	8.2 – 52.3 (30.0)	0.24 –4.44 (1.83)	12.4 – 430 (103)
^a^ Linggi River, Negeri Sembilan (n = 8)	–	–	26 – 78 (42)	13 – 74 (30)	15 – 28 (24)	33 – 92 (56)	–	35 – 135 (78)
^b^ Terangganu River, Terengganu (n = 42)	0.83 – 23.09 (9.48)	0.35 – 0.52 (0.44)	–	–	–	0.051 – 124.9 (32.1)	–	–
^c^Kerteh River, Terengganu (n = 9)	–	4.0 – 5.0 (4.3)	13 – 67 (33.7)	6.3 – 20.0 (11.2)	7 – 24 (11.2)	11 – 25 (15.5)	–	16 – 62 (44.7)
^d^Kelantan River, Kelantan (n = 26)	–	(1.82)	–	(6.47)	–	(20.82)	–	(18.67)
^e^Juru River, Penang (n = 7)	–	–	–	2 - 144	–	0 - 117	–	2 - 483
^f^Langat River, Selangor (n = 10)	12.4 – 27.3 (17)	3.0 – 37.9 (12.1)	11 – 73 (29)	–	–	–	0.82 – 5.0 (3.1)	71 – 374 (154)
^g^Klang River, Selangor (n = 21)	–	0.57 – 2.19 (1.54)	–	10.6 – 63.0 (37.1)	5.9 – 24.5 (16.3)	24.2 – 64.1 (47.9)	–	31.9 – 272 (163)
^h^Pelepah Kanan River, Kota Tinggi, Johor. (n = 15)	98 – 279 (149)	–	5 – 30 (17)	58 – 259 (117)	0 – 9.0 (5.3)	10 – 54 (34)	–	44 – 100 (61)
^i^Canadian-FSQGs – TEL value	5.9	0.60	37.3	35.7	18	35.0	–	123
^i^Canadian-FSQGs – PEL value	17.0	3.50	90.0	197	36	91.3		315
^j^Consensus-FSQGs – TEC value	9.79	0.99	43.4	31.6	22.7	35.8	–	121
^j^Consensus-FSQGs – PEC value	33.0	4.98	111	149	48.6	128	–	459

^a^ [12], 1990; ^b^ [27]; ^c^[28] ; ^d^[29]; ^e^[30]; ^f^[31]; ^g^[32]; ^h^[33]; ^i^ [34]; ^j^[11]; value in parentheses = mean concentration; TEL – Threshold Effect Level, PEL – Probable Effect Level; TEC - Threshold Effect Concentration; PEC – Probable Effect Concentration.

## Conclusions

The enrichment factors and degree of contaminations showed that the sediments collected from the Linggi River were polluted with toxic elements As, Cd, Pb, Sb, and Zn. The source of As, Cd, Pb, Sb, and Zn pollution were originated from industries. Amongst the elements analysed, As showed high EF and C_f_ values in most of the sampling stations. The mean As concentrations of Linggi River sediments showed higher concentration than values from the guidelines of Canadian-FSQGs - PEL (17.0 mg/kg) and Consensus-FSQGs – PEC (33.0 mg/kg). The high concentrations of toxic elements such as As and Cd than those of the PEC-FSQGs could result in an adverse effect on the benthic organisms and marine ecology. The results of the assessment of Linggi River sediments obtained from this study will provide vital information that can be used for future comparison. Information from the present study will be useful to the relevant government agencies and authorities in preparing preventive actions to control direct discharge of toxic elements and other pollutants from industries, agro-based activities and domestic wastes into the rivers.
